# Correction: Conformity and tradition are more important than environmental values in constraining resource overharvest

**DOI:** 10.1371/journal.pone.0334112

**Published:** 2025-10-08

**Authors:** Glenn Wright, Carl Salk, Piotr Magnuszewski, Joanna Stefanska, Krister Andersson, Jean Paul Benavides, Robin Chazdon, Michał Pająk

Michał Pająk is not included in the author byline. Michał Pająk should be listed as the eighth author and affiliated with Department of Mathematical Economis, Faculty of Economics and Finance, Wroclaw University of Finance and Business, Wroclaw, Poland. The contributions of this author are as follows: Conceptualization/Investigation/Methodology/Project administration/Software.

## References

[1] Wright G, Salk C, Magnuszewski P, Stefanska J, Andersson K, Benavides JP, et al. Conformity and tradition are more important than environmental values in constraining resource overharvest. PLoS One. 2023;18(2):e0272366. doi: 10.1371/journal.pone.0272366 36730172 PMC9894460

