# Supplementary material for: Conformity and tradition are more important than environmental values in constraining resource overharvest
Source: PLoS One. 2023 Feb 2;18(2):e0272366. doi: 10.1371/journal.pone.0272366 (PMC9894460; doi:10.1371/journal.pone.0272366)
Supplement: S1 File — (DOCX) [file pone.0272366.s001.docx]

# Introduction

This document explains how to moderate the forest management activity. It is for use by the moderator, not the participants. All information that should be read to the participants is in italics.

# Activity and rules

## Inventory

At the beginning of the activity, each participant will be given:

1. A badge – a picture of an animal and a number representing the participant within the activity.
2. A decision box - a box with an identifying animal picture and number on the outside, a number on the inside (these should be the same as the animal/number on the badge) and four slots within:
   - Harvesting slot - where the participant puts completed harvesting forms and the moderator puts income from harvesting.
   - Monitoring slot - where the participant puts completed monitoring forms and tokens to pay for monitoring, and where participants can find monitoring results from the moderator
   - Sanctioning slot - where participants put their sanctioning forms and tokens to pay for sanctioning
   - Storage compartment – a place where participants can put whatever they want

For the practice rounds, each box should contain:

- Harvesting forms (5) for the 5 practice rounds (later changed into regular harvesting forms)
- Monitoring forms (15) for the 5 practice rounds (later changed into regular monitoring forms)
- Sanctioning forms (15) for the 5 practice rounds (later changed into regular sanctioning forms)
- Three practice income tokens. The tokens used for practice should be different from those in the regular rounds.

## Forms and cards

There are various forms that are used by the participants. There are separate forms for practice rounds and the regular activity that differ both in terms of content and background color. There is also a moderator’s sheet that is used by the moderator to record all decisions and results during the regular activity. The moderator’s sheet will be described in detail in section 6.4.

The forms presented below use animals to identify the participants. They are based on set of animals for use in Uganda. There is separate set of animals that should be used in Bolivia.

S1 Fig. Harvesting form

The harvesting form is used to inform the moderator about a participant’s harvesting decision. It is used once per round. A participant uses lines or a number to indicate how many trees he/she wants to harvest.

S2 Fig. Monitoring form

This form is used by a participant to inform the moderator who he/she would like to monitor. The moderator uses the same form as a monitoring report for the participant.

This form is different for each participant. It is a different color than the other forms but is the same color for all participants). When a participant wants to monitor another participant(s), he/she must mark the participant(s) to be monitored. All of this can be done with a single sheet as the participant can mark more than one animal.

The monitoring report returned to the participant by the moderator is the same form submitted by the participant. The moderator writes the round number in the space by the picture of the man looking at the footprints (practice rounds should be marked with a P plus the number of practice round. Rounds of the regular activity should be marked with a round number). The moderator then fills out the form with the harvesting decisions of each participant who was monitored below the appropriate animal. Harvesting decisions should be represented by a number of lines, rather than a number, if the participants are not familiar with numbers.

S3 Fig. Sanctioning form

The sanctioning form is used only by the participants. It is different for each participant and has a different color than the other forms (but is same color for all participants). After it is filled out and sanctions are applied, the moderator shows and reads it to the entire community.

Participants write a number or lines to indicate how many tokens are used to sanction each other participant.

S4 Fig. Debt card

The debt card is filled out by the moderator. The number (or lines) represents the current debt of the participant.

## Forest

The forest is represented by a 10x10 square composed of 100 forest tokens. Each token represents a tree. Trees are always taken from or added to the same side of the board to make it easier for participants to estimate the state of the forest and for the moderator to calculate forest regeneration. The forest should be placed on the prepared board so participants can easily see how much of the forest remains.

In each round, the moderator will take away all harvested trees and after that add new trees representing natural regeneration. The forest regeneration rate depends on the number of existing trees. Every round two new trees will be added to the forest for every full row of 10 trees present. When there are fewer than 10 trees in the forest (less than one full row) the regeneration is two trees per round.

## Finances

Money is represented by tokens. Participants earn them by harvesting trees. After the activity, tokens will be exchanged for shillings based on tokens that are in the participant’s possession.

Participants are free to use their tokens however they wish during the activity. They are free to transfer them to other participants and use them as part of institutional arrangements. Financial flows will not be tracked by the moderator. If any institutional arrangement connected with use of the tokens emerges, it should be noted on the observation form.

If a participant has a debt, it will be reduced from income in subsequent rounds or at the end of the activity. Participants cannot be forced to repay debts from tokens in their possession. Still, debt must be taken into account when tokens are exchanged for shillings at the end of the activity by subtracting it from tokens that are in the participant’s possession.

The activity can be divided to two separate parts - practice rounds and ’real’ rounds. Tokens earned during practice rounds will not be counted toward participants’ results; only those earned during the actual activity should be changed into shillings. Also at the beginning of the actual activity each participant will receive an endowment of 3 tokens so they will be able to do monitoring and sanctioning in the first round if they wish. There are different income tokens for practice rounds and regular activity.

## Forest harvesting

In each round, each participant decides how many trees to harvest. The maximum allowed harvest per individual depends on the state of the forest - it will decrease with depletion of the forest. For each tree harvested, the participant will receive two tokens.

**S1 Table. Allowable harvest**

| Trees in forest /  Percentage of initial forest | Individual maximum harvest level |
| --- | --- |
| 48 – 100 | 6 |
| 40 – 47 | 5 |
| 32 – 39 | 4 |
| 24 – 31 | 3 |
| 16 - 23 | 2 |
| 8 - 15 | 1 |
| 0 - 7 | 0 |

Deforestation leads to lower maximum harvest levels and therefore to lower potential income.

## Cost of living

In each round the participants must pay for their costs of living. The moderator gathers tokens as participants hand in their result boxes so paying costs of living is distinguished from losing tokens from sanctions.

If a participant cannot pay the cost of living it is treated as a debt that should be repaid in subsequent rounds..

Costs of living are always collected for the round that is currently in progress.

## Monitoring and sanctioning

Monitoring and sanctioning are conducted with monitoring and sanctioning forms (which have different colors so they can be easily distinguished). Participants have to pay at least one token to conduct either of these activities.

Each participant can monitor any other participant. A report on a participant’s harvest level is provided for a fee of one token. Information about the results of monitoring is given only to the participant who paid for the monitoring.

Other participants can be sanctioned. This also requires that a fee be paid. One token spent on sanctioning another participant will result in the sanctioned participant losing two tokens from his/her income. Information about sanctions is shown and read to the entire community after the sanction is applied.

If sanctions exceed the participant’s income, the participant receives no income and goes into debt, to be repaid in subsequent rounds.

## Using decision boxes

The decision boxes have four separate compartments. Three are connected with types of decisions, while the last one can be used by participants for storage. In each round, participants make decisions by filling out forms and putting them in the appropriate slot.

When decision boxes are handed back to the participants, the moderator uses the slots to inform them individually of their results. Tokens (or debt forms if necessary) are put into the harvesting slot after removing the harvesting form. Monitoring forms are filled out and returned to the monitoring slot. Sanctioning forms are removed from the box and given to the moderator.

The moderator has no access to the storage slot. Even if a participant is in debt and there are tokens inside the storage box, they cannot be taken. If participants leave old monitoring reports in the monitoring slot, the moderator should put those into the storage slot. Also, other misplaced forms should be moved into storage by the moderator.

## Communication and creating institutions

Participants are free to create their own institutions. No rules impose any restrictions on creating institutions. Further, open communication is allowed regardless of the topic discussed. Observation forms are used to analyze communication and the emergence of institutions.

Participants can inform each other about their decisions and results but they are forbidden to show each other their forms. This rule is given to participants in their instructions. If participants do not follow this rule, the moderator should stop the activity for moment and all participants should be reminded how the rule works.

## PES treatment

Half of the activities in the field (one per site) should be conducted with a PES treatment. In activities without PES, participants can earn money only by harvesting trees. In the PES treatment, participants receive income for harvested trees and for intact forest at the end of the activity. Each participant will get 4 tokens (worth in total of 300 shillings) for each row of trees that was left in the forest.

In the field both activities should be conducted in alternating order.

## Activity flow

The activity lasts for 15 rounds. Each round involves participants analyzing current information and making decisions. There is no restriction on the length of a round - the moderator should delay the return of decision boxes so as not to interrupt the participants’ discussion. The following table illustrates the order of events within a round. The moderator is the person who explains the activity to the participants and gives them feedback about the forest. The cashier is the person who manages the boxes and processes the participants’ decisions.

**S2 Table. Activity flow**

| **Moderator** | **Cashier** | **Participants** |
| --- | --- | --- |
|  | Calculate the total harvest and participants’ results |  |
|  | Prepare monitoring reports |  |
|  | Apply sanctions |  |
| Give participants their results and monitoring reports |  |  |
| Remove harvested forest from the board |  |  |
| Add regeneration to the forest |  |  |
| Inform the community about sanctions that were applied |  |  |
| Remind participants about open communication |  |  |
|  |  | Check results and monitoring reports |
|  |  | Decisions about harvest |
|  |  | Decisions about monitoring |
|  |  | Decisions about sanctioning |
|  |  | Give decision boxes to the moderator |
| Collect decision boxes |  |  |
| Collect cost of living |  |  |

Before the first round, the cashier has to remove all practice items from the boxes and put new forms into them. After the boxes are prepared, they are given to the moderator.

The moderator gives the boxes to the participants and informs them about the state of the forest.

Each round ends when participants hand in their decision boxes to the moderator and he collects their costs of living. The observers should begin a new round’s observation sheet when this happens.

## Room layout

Participants should be placed in a circle or square where each participant can easily communicate with everyone else. It is necessary to leave some distance between participants so they can conceal the contents of their decision boxes from others. The forest should be placed inside of the circle so that all participants can easily see it. The cashier’s table, where all results will be calculated, should be outside of the circle, far enough away so that participants will not be able to see the moderator processing decisions.

Information about sanctions should be visible to all participants. After announcing sanctions, the moderator can put the sanctioning forms next to the forest. They should remain there for one round and should be replaced with new sanctions or taken off if no new sanctions are issued.

# Presentation and explanation - instructions for the moderator

The practice rounds give participants a chance to test all rules and features before the actual activity starts. The moderator also will be able to check whether participants understand the rules.

Participants’ decisions in practice rounds are guided by the moderator. Rather than making decisions, the participants follow the instructions of the moderator, and the forms are checked to see if they are filled out correctly.

Different features are introduced in subsequent rounds. In the first round, participants are asked to pay costs of living and harvest, in the second they pay costs of living, harvest and monitor, in the third they pay costs of living, harvest and sanction, and in the fourth they pay costs of living, harvest, monitor and sanction. In the fifth, they pay costs of living, and harvest, monitor and sanction as they wish. Appropriate forms and three practice tokens are given to the participants by the moderator with the instructions for the activities. Also, there are example forms that should be shown to participants. They are like the harvesting, monitoring and sanctioning forms, but are larger. The moderator should use them to explain how to fill out the forms.

After each round, all forms will be checked for errors by the moderator. If they are filled out correctly, they are handed in to the cashier. While the forms are processed, the moderator gives participants additional instructions.

When an incorrectly filled form is found during the processing, it should be returned to the participant to be corrected after giving participant an explanation of the mistake. This should be done only after the moderator is finished giving participants additional information while the cashier processes decisions. After each part of the oral presentation, there is time for participants to ask questions. If they do not understand an instruction, it should be rephrased, without adding any new information. If participants ask questions about activity elements that will be explained later they should be informed that it will be mentioned later.

After the practice rounds, the forest will return to its original state (100 trees) and no money earned will be transferred to the actual activity. All participants will be given endowment of 3 tokens at the beginning of actual activity so they will be able to pay costs of living and if they wish, monitor and/or sanction others during the first round.

# Activity introduction protocol

This protocol introduces participants to the activity. There are five practice rounds.

### Introducing decision boxes

This information should be presented when participants first receive their boxes.

MODERATOR:

*Thank you for coming. We are part of a group of researchers interested in forest management.*

*My name is XXXX, and during today’s activity, I will be the moderator who explains what we will be doing. I am accompanied by three additional people. YYYY is the cashier, and ZZZZ and WWWW will take notes on what happens during the activity.*

*Today we would like you to take part in an activity. In this activity you will be members of a small community that uses forest resources to earn money and satisfy its basic needs. All of your earnings during this activity will be converted into shillings after we finish. You will earn a minimum of 5000 shillings for participating, and additional money depending on your decisions.*

*Remember that this activity begins and ends here in this room.* *All actions you take as participants and members of this community shouldn't have any effect on your actions after the activity.*

*Each of you received box and a badge with a picture of an animal on the front. As you learned during the surveys, within the activity you will be identified with this animal. Now I would like each one of you to show your pictures to the other participants and tell them what animal is in the picture. [time for participants to show picture on their badges and boxes]*

*Please open your boxes. Please note the pictures that function as labels in three of the four compartments. Their function and meaning will be explained shortly.*

*Is everything clear? Do you have any questions?*

### Introducing the forest

The forest should be set up before starting this section. The moderator should also possess tokens representing trees and some income tokens as they will be needed during here.

MODERATOR:

*During this activity you are a community that uses forest resources to earn money and satisfy its needs.*

*[Moderator shows participants the forest]*

*This is your forest. At the beginning of the activity, it has 100 trees. During each round you will be able to harvest trees to earn money. During the activity, the money is represented by tokens.*

*Is everything clear? Do you have any questions? Can we proceed?*

MODERATOR (NON-PES TREATMENT):

*For each tree you extract, you will earn 2 tokens, each of which is worth 100 shillings.*

*[The moderator shows the participants 2 practice round tokens]*

MODERATOR (PES TREATMENT):

*For each tree you extract, you will earn 2 tokens, each of which is worth 75 shillings*

*[The moderator shows the participants 2 practice round tokens]*

*In addition, at the end of the activity an external organization that values conservation will offer a bonus for the trees remaining in the forest.  Each participant will earn 4 tokens (equivalent to 300 shillings) for each complete row of trees remaining in the forest.*

MODERATOR:

*First, we will have some practice rounds that will help you learn how the activity works. You will earn shillings once we begin the real activity; you don’t need to worry about your earnings during the practice rounds as these will not count toward your final earnings. During the practice rounds we will use tokens that are different from those in the real activity. At the end of the practice, the forest will return to its original state with 100 trees.*

### Introducing forest harvesting and costs of living

*You can earn money by harvesting trees.*

*[the moderator shows the example harvesting form to the group]*

*The harvesting form looks like this, and has a drawing of a person cutting a tree.*

*You harvest trees from the forest with this form by writing a number or just a number of lines that indicates how many trees you want to harvest in each round. The completed form should be put into the harvesting compartment – it is labeled with a picture of a man cutting a tree.*

*Each tree the groups harvest will be removed from the forest.*

*Is everything clear? Do you have any questions?*

MODERATOR:

*In each round you will have to pay a cost of living of one token. This cost pays for things like your food, water and home. In each round, I will ask each of you to give me one token.*

*Is everything clear? Do you have any questions?*

After the moderator answers questions or there were no questions, the practice rounds may begin.

MODERATOR:

*Now we are going to do some practice rounds. In these rounds, I will tell you how many trees to cut and what else to do. In the real activity, how many trees you cut and your other decisions will be your choices, and you won’t have to share them with anyone unless you want to.*

*Each of you has your decision box in front of you. Please fill out a harvesting form for practice.*

*Because the forest has 100 trees, the maximum number that each person can harvest is 6. Please fill out your forms to harvest 6 trees and put them into harvesting slot in your decision box.*

*[The moderator waits for participants to fill their forms and verifies that they are done correctly and put in the correct compartment before collecting the boxes]*

If any form is filled out incorrectly, follow the procedure in section 3.

MODERATOR (NON PES):

*This is your forest and there is no government or external authority that makes or enforces rules about forest use. Your decisions about how many trees to extract will affect the state of the forest.*

MODERATOR (PES VERSION):

*This is your forest and there is no government or external authority that makes or enforces rules about forest use. Your decisions about how many trees to extract will affect the state of the forest.*

*However, there is an external organization that values conservation that will pay you a bonus for trees remaining in the end of the activity.*

*MODERATOR*

*I asked each of you to cut six trees. This means that in total, the group cut 48 trees.*

*[The moderator removes 48 trees from the forest]*

*The forest will grow, just like real forests. In each round, two new trees will grow for every full forest row of 10 trees that remains in the forest.*

*After your harvesting, there are 52 trees in the forest. Because there are 5 full rows of trees, 10 new trees will grow, for a total of 62 trees.*

*[Moderator adds 10 tree tokens to the forest]*

*Is everything clear? Do you have any questions?*

Fill the boxes with the correct number of tokens and return them to the participants.

MODERATOR (NON-PES TREATMENT):

*You have received 12 tokens in your box for the trees you cut.*

MODERATOR (NON-PES TREATMENT):

*You have received 12 tokens in your box for the trees you cut.*

*Additionally, remember that at the end of the activity, an external organization that values conservation will pay a bonus for the trees that remain in the forest. Each participant will earn 4 tokens (equivalent to 300 shillings) for each complete row of trees that remains in the forest.*

MODERATOR:

*You don’t have to tell anyone how many tokens you earned. If you wish, you may share that information, but you don’t have to.*

*Please don’t show anyone your forms. If you wish to inform others about your decisions, please tell them verbally.*

*It is not possible for the forest to have more than 100 trees. If it reaches this state, all available land is covered by forest and no more trees will be added.*

*It is possibly that you cut the entire forest and less than no trees remain. In that case it will still regenerate, but only two new trees will appear per round.*

*Is everything clear? Do you have any questions?*

### Introducing monitoring

MODERATOR:

*All your decisions are private. You do not have to share this information with others if you do not want to. Sometimes it might be useful to know how much other participants have harvested. If you want, you can ask others about their harvesting decisions. The other person can share this information, decide not to reveal it or even lie.*

*You can find out for sure how much other participants harvested by monitoring.*

*If you wish to monitor, you may spend one token per person monitored.*

*In this practice round please monitor the person seated to your right.*

*[Moderator shows the example monitoring form to the group and explains it]*

*You do monitoring by putting a mark under the animal representing the participant you want to monitor, in this case, the participant seated to your right.*

*[The monitor should tell each participant who is seated to the right and how to fill out the form (do this one by one)]*

*When you are finished, put the form into the monitoring compartment. This compartment is labeled by a picture of a man following footprints.*

*After filling out the form you need to pay for monitoring which costs one token per person monitored. Now pay for your monitoring. You are monitoring only one person so place one token in the monitoring slot.*

*After all information is processed you will get a report that says how many trees were cut by the monitored participant.*

*For practice, you will also harvest some trees. There are still 62 trees in your forest so you can harvest up to 6 trees. For now, I will ask each of you to harvest 3 trees.*

*After you have filled out your monitoring and harvesting forms, please hand me your boxes and don’t forget to pay your costs of living.*

*Is everything clear? Do you have any questions?*

Boxes are taken by the moderator. If they were not filled out correctly, the procedure from section 3 should be used. While decisions are processed, the moderator should give additional instructions.

*MODERATOR:*

*When you get monitoring information you can share it with others or keep it to yourself. You will decide what to do with it.*

*Remember that you will only get information about harvesting by the participant to your right from the round in which you did monitoring. If you want to know what he’ll do next, you need to monitor again.*

*It is also possible to monitor more than one person. All you have to do is to make a mark under each participant you want to monitor and pay one token per person monitored If you have enough tokens, you can monitor everyone in the community if you wish. On the other hand, you do not have to monitor anyone if you do not want to.*

*Is everything clear? Do you have any questions?*

*Each of you cut 3 trees, so the harvest for the entire group was 24 trees.*

*[The moderator removes 24 trees]*

*There are 38 trees remaining in the forest, so there are 3 complete rows, meaning 6 new trees will grow.*

*[The moderator adds 6 trees]*

*Now there are only 44 trees in the forest. This means that it is harder to find suitable trees to harvest, so in the next round, you are allowed to harvest no more than 5 trees. At the beginning of each round, you will be told the maximum harvesting limit. When the forest is smaller, the harvesting limit goes down.*

*[The moderator returns the boxes.]*

MODERATOR:

*You have received your results including monitoring reports. Each of you should receive tokens from harvesting, in this case 6 tokens. You also received a monitoring report saying how many trees the person seated to your right cut.*

*[Moderator shows the participants a completed monitoring form example]*

*Is everything clear? Do you have any questions?*

### Introducing sanctioning

MODERATOR:

*Last time you learned how to monitor others. If you think that other participants are acting unfairly, you may punish them. Sanctioning other participants will also require payment of tokens. If you decide to punish someone, you will have to pay some number of tokens and the person sanctioned will lose twice as many tokens. If you decide to pay 2 tokens to sanction someone, they will lose 4. If you pay 3 to sanction another player, they will lose 6. You can decide to sanction with the any number of tokens, as long as you have enough to pay for it.*

*To sanction a participant you have to fill out a sanctioning form. You have to write how many tokens you would like to pay to sanction that person with.*

*Please sanction the person to your left with one token. This means writing ‘one’ under the picture of the animal representing the participant you want to sanction. Fill out the form now.*

*Now, put this form inside the sanctioning compartment – it is labeled by person shouting at someone.*

*The last thing you have to do to successfully sanction is to put one token inside the sanctioning compartment.*

*Put the income token to the sanctioning compartment now.*

*In this round you will also harvest. More than half of the forest is gone, so harvest only one tree.*

The boxes are collected by the moderator. If the forms were not filled out correctly, follow the procedure from section 3. While decisions are checked and processed, the moderator should give additional instructions.

MODERATOR:

*If you decide to sanction more than one person please write how many tokens you want to sanction each person with and provide tokens in the sanctioning compartment. For example, if you want to sanction one person for with one token and a second person with two tokens, you simply write the number ‘1’ under the first person’s animal, and ‘2’ under the second person’s animal and put three tokens in the sanctioning compartment.*

*It is your decision whether to punish someone. You can sanction anyone for any reason - the sanction will be applied as long as you provide enough tokens. You can sanction the entire community if you want to as long as you have enough tokens. You do not have to sanction anyone if you don’t want to.*

*Unlike monitoring, information about sanctions will be available to everyone. After each round I will announce all sanctions. This means that everyone will know who punished whom.*

*Is everything clear? Do you have any questions?*

*During this round, 8 trees were cut from the forest.*

*[Moderator removes 8 trees]*

*This means that 36 trees remain. Because there are 3 full rows, 6 new trees will appear.*

*[Moderator adds 6 trees]*

*There are now 42 trees in the forest. The maximum possible harvest is only 5 trees.*

*Now I will inform whole community about sanctioning.*

*[Moderator reads out all sanctioning reports and puts them where everyone can see them]*

*Each of you sanctioned the person to your left. This means that X sanctioned Y with two tokens, that Y sanctioned Z with two tokens, etc.*

When decision boxes are given back to the participants the moderator can go on with the instructions.

MODERATOR:

*You all have your results now. You earned some income tokens.*

MODERATOR:

*If you don’t have enough tokens to pay your costs, you acquire a debt that is deducted from your income in the future.*

*[The moderator shows the participants a debt card.]*

*Each token that you owe is represented by a line or number on the debt card. Your debt will accumulate in case you are not able to pay cost of living or are sanctioned beyond your earnings in multiple rounds.*

*Remember that if you end the activity with a debt you will still receive a payment of 5000 shillings for your participation, but you won’t receive any additional payment.*

*If you are in debt because of sanctioning or not being able to pay cost of living this debt will be taken from income in future rounds. All debt is recorded and will be taken into account when we calculate how many shillings you earn from the activity.*

### Practice round with harvesting, monitoring and sanctioning.

MODERATOR:

*We are going to do another practice round in which you will harvest trees, monitor a participant and sanction a different participant.*

*Please take a harvesting form and fill it in to extract 1 tree. Put this form in the corresponding slot.*

*[The monitor explains to each participant who they are going to monitor and sanction, one by one.]*

*In this practice round you will monitor the participant opposite you. Take a monitoring form and fill it out to monitor this person. Place the completed form and one token in the corresponding slot.*

*In this practice round, you will sanction the second person on your right with two sanctions. Take a sanctioning form, fill it out, and place it in the corresponding slot with two tokens.*

The boxes are collected by the moderator. If the forms are not filled out correctly, follow the procedure in section 3. While the decisions are being processed, the moderator should give additional information.

*[The moderator should encourage the participants to discuss the activity]*

MODERATOR:

*During this round, 8 trees were cut from the forest.*

*[moderator removes 8 trees]*

*This means that 34 trees remain. Because there are 3 full rows, 6 new trees will appear.*

*[The moderator adds 6 trees]*

*There are now 40 trees in the forest.*

*Now I will inform the community about sanctioning.*

*[The moderator reads out all sanctioning reports and puts them where everyone can see them]*

*Each of you sanctioned the person to your left. This means that X sanctioned Y with two tokens, that Y sanctioned Z with two tokens, etc.*

Ones the boxes are returned to the participants, the moderator can continue with the instructions.

*You just received your boxes with your results. Since you cut 1 tree and sanctioned somebody with 2 tokens, you have a debt form in your box.*

*You also got the results from the monitoring.*

### Free practice round with harvesting, monitoring and sanctioning.

MODERATOR:

*We will now have a final practice round in which you will harvest, monitor and sanction, but this time, instead of me telling you how much to harvest and whom to monitor and sanction, you will decide on your own.*

*Because the forest now has 40 trees, the harvesting limit for this round is 5 trees each. Please fill out your harvesting form with the number of trees you would like to cut. Also, fill out a monitoring form and a sanctioning form. In this practice round, you can choose who you want to monitor and sanction, and even monitor or sanction more than one person.*

The boxes are collected by the moderator. If the forms are not filled out correctly, follow the procedure from section 3. While the decisions are being processed, the moderator should give additional information.

*[The moderator should encourage dialogue.]*

*[When ready, the moderator should remove the correct number of trees, and place new trees in the forest.]*

*There were some sanctions in this round:*

*P sanctioned Q, etc.*

*[For each penalty, the sanctioning form should be shown to the participants and placed somewhere visible to the participants.]*

When the boxes are returned, the moderator should continue with the instructions.

*[Return the boxes to the participants.]*

*You just received your results for this round. Look inside your boxes. Based on your decisions, do you see what you expect? Are there any surprises?*

### Finishing the practice rounds

MODERATOR:

*This was the last practice round. Your earnings during the practice rounds won’t be taken into account for your payment at the end of the activity. At the end of real activity all your earnings from harvesting the forest would be changed into shillings.*

*MODERATOR (PES TREATMENT):*

Remember that an external organization *will give you a bonus depending on the state of the forest. Now there are 40 trees in the forest. If this was the last round of the regular activity, each of you would get 4 tokens for each full row of trees that remained in the forest. Each of you would be paid an additional 16 tokens, worth 1200 shillings, since there are 4 full rows of trees.*

*MODERATOR:*

*The activity we have just done was for practice, so you haven’t earned or lost any money. You will earn shillings during the actual activity that will start in a moment. Please hand me your decision boxes now. Tokens and all completed forms will be removed to prepare you for the activity. Also, each of you will receive 3 tokens at the beginning of the activity.*

*[Boxes are collected and all unnecessary items are removed. It is important to check if all tokens from the presentation have been collected and 3 tokens are put in each participant’s box.]*

### Recapitulating activity flow

MODERATOR:

*During each round you will decide if you want to cut trees, and if yes, how many you want to extract. You will also decide if you want to monitor or sanction other participants.*

*Processing your decisions will take some time. If you would like, you may use this time to talk among yourselves. Feel free to talk about whatever you like at any time during the activity.*

*You may decide to discuss the state of the forest, share information, or make rules about the use of the forest. These can be about different ways of managing the forest. In your community, you many have some rules like this about the real forest.*

*Remember that if you want to talk about your decisions and results, you are free to do so as long as you do not show your forms to other participants.*

*If you don’t want to talk, you may remain silent. It is up to you whether you talk or not.*

*This forest belongs to your community and no one from outside this group may use it. Your decisions will shape the future of the forest. Remember that there is no government or central authority to govern this forest. Your community is responsible for this forest and only your decisions will affect its condition*

*The activity will last 15 rounds and you may decide as a group how you want to manage the forest, therefore I invite you to talk while we are preparing your boxes.*

*Here you have the forest with 100 trees ready for the first round.At the beginning of the first round the forest has 100 trees. In this round, you may cut up to 6 trees.*

## Introducing the first round

At the beginning of the first round the forest should be returned to its original state with 100 trees.

MODERATOR:

*I am now handing out your boxes in which you will find all the forms you need for the activity and three tokens.*

*Please make your decisions about how many trees to cut, and whether you want to monitor or sanction. Now you can discuss whatever topic you consider important, and when you are ready, make your decisions by filling out the corresponding forms. When this is done, please hand me your boxes along with one token for cost of living and your decisions will be processed.*

*In the practice rounds, I told you who to monitor and sanction, but that doesn’t mean you should always do these things to those people. You are free to sanction and monitor whomever you wish, or not to do this to anyone.*

*How is the forest doing now? How will you manage it?*

# Instructions for the moderator

## Instructions for the beginning of every round

Each time a new round begins the moderator should inform participants about sanctioning and changes in the forest.

MODERATOR:

*During this round X trees were harvested.*

*[Moderator takes X trees from the forest]*

*There are Y trees left. This means that there are Z full rows of trees so 2*Z new trees will grow.*

*[The moderator adds new trees to the forest]*

*Now there are W trees in the forest. In this round you will be able to harvest up to T trees. Now you have time to discuss anything that you feel is important and make your decisions.*

*After you make your decisions please hand me the boxes so your information can be processed.*

*[If there was sanctioning]*

*There was some sanctioning in this round:*

*P sanctioned Q.*

*[Each sanctioning form is shown to all participants and put in a place visible to all]*

*[After giving back all boxes]*

*You have received your results from the previous round. If you harvested trees you received tokens for your decisions.*

## Cashier’s sheet

The cashier’s sheet is a piece of paper that is used for recording participants’ decisions in each round to capture the activity’s mechanics. Participants are represented both by numbers and pictures so the moderator’s form is country dependent (here the version for Uganda is presented). It is divided into four sections.

S5 Fig. Cashier’s sheet section 1

The first section is for recording information about the location and round. Then the cashier records the harvesting decisions of the participants. TOTAL is used to calculate total harvesting for the round - the number of trees that were cut from the forest.

The forest section is for calculating changes in the forest. This serves as a record of changes during the activity, and as a check that the moderator has correctly changed the forest.

S3 Table. Cashier’s sheet section 2

| Trees at end of last round |  |
| --- | --- |
| Total harvest | - |
| Remaining after harvest | = |
| Full rows x2 | + |
| Trees after regrowth | = |

S6 Fig. Cashier’s sheet section 3

The monitoring section is used only when monitoring takes place. The cashier writes just the numbers of the participants who were monitored by each participant in each column.

S7 Fig. Cashier’s sheet section 4

The sanctioning section is a matrix. If a participant sanctions others he/she must be found in the column on the left. Information about who was sanctioned must be written in the row corresponding to the participant who is doing the sanctioning. For each participant who is sanctioned, it should be noted how many tokens were used for punishment.

The ‘Debt’ row records participants’ accumulated debt from the previous round (if there were any) and should be a negative number.

After all decision boxes are given back to participants, the moderator should collect costs of living. When a participant is able to pay costs of living, nothing should be written in the ‘Costs of living’ row. If they are not able to pay, write ‘-1’ in that cell.

The ‘Sanctions*2’ row is filled out by summing all sanctions on each participant and multiplying by 2; it should also be a negative number.

The ‘Income’ row is a participant’s income from harvesting forest resources. It should be between 0 and 12.

The ‘Cash’ row is the sum of the rows that are above (Debt + Cost of living + Sanctions*2 + Income). If it is a positive number, the participant earned tokens and they should be placed inside the decision box. If it is a negative number, the participant should be given a Debt card with the appropriate number.

## Processing information

Information processing can be split into three parts.

First, the moderator informs the cashier about collected costs of living. If a participant cannot afford costs of living this this information should be given to the cashier and the moderator should update a participant’s debt card. A new debt card should be given to the participant with indicating their current debt. Old debt cards should be taken from the participant by the moderator.

The cashier has to indicate whether those costs were paid. He/she also checks the harvesting forms and writes down the decisions on the cashier’s form. Harvesting decision forms must be taken out of the box and replaced by tokens. Sanctions should be noted on the cashier’s form, and the sanctioning form should be removed from the box. After processing all the boxes in this way, the cashier should calculate sanctions for each participant.

The second part is monitoring and sanctioning. If there is a completed monitoring form, the cashier should take it out of the box, note this on the cashier’s form and fill out a monitoring report remembering about to add the round number to the monitoring report. The monitoring report should be put inside the monitoring compartment. Then the cashier should check if any sanctions were issued for the current participant. If there were any, tokens should be taken out and if it is necessary, a debt card should be placed inside the box.

The cashier should have enough space so all boxes can be opened at the same time, which should speed up calculations. Also, the workspace for the cashier should be outside of the participants’ view.

If a form is filled out incorrectly or there are not enough income tokens for sanctioning/monitoring the cashier should give it back to the moderator, so he can ask the participant to clarify his/her decision. Only incorrect forms should be returned to participants to minimize interference in processing other boxes.

Handing the boxes back to the participants is done once the cashier has finished calculating participants’ results from the round that just ended.

## Debriefing

MODERATOR:

*This was the last round of the activity.*

*During this round X trees were harvested.*

*[Moderator removes X trees from the forest]*

*There are Y trees left. This means that there are Z full rows of forest so 2Z new trees will grow because of regeneration.*

*[Moderator adds new trees to the forest]*

*Now there are W trees in the forest. As this was the last round of the activity, this is the final state of the forest.*

*[If there was sanctioning]*

*There was some sanctioning this round:*

*P sanctioned Q.*

*[With each sanction the sanctioning form is shown to all participants and put in a place visible to all]*

*You have received your boxes with your results. If you decided to harvest you have received your tokens.*

MODERATOR (PES TREATMENT):

*In addition to your income form harvesting trees,* an external organization that values conservation will offer a bonus for the trees remaining in forest, thus *each of you will also get 4*V tokens, as there are V full rows of trees remaining in the forest.*

MODERATOR:

*Now please hand in your boxes. While the cashier calculates your earnings, we will ask you to complete a final survey. When you have completed the survey, we will call you one by one to pay your earnings.* [after the boxes are taken]

*Once again thank you for taking part in this activity today. Please remember that all that happened here was only an activity. Your decisions, actions and animals representing you were just part of the activity and have nothing to do with who you are in real life. If you have any comments or questions regarding the activity and what we are doing here feel free to ask them now.*

Complete post-game surveys. When one person is paid, tell the next that it is their turn.

MODERATOR:

*Now you are free to go. Thank you once again for coming and please leave your boxes, your animal representations and any other material used during the activity. Thank you very much.*

## Returning final results

The moderator collects the boxes after the last round of the activity and the cashier calculates the results of all the participants while the other three team members do the surveys. Once the surveys have been completed, the participants should be called one by one to receive their earnings. The money (in cash) should be given directly to each participant. At the same time, each participant should put his/her initials on a receipt.

# Summary of Materials Needed

Listed below is the number of printouts needed for trial rounds and 15 rounds of the regular activity.

- 8 decision boxes with a different animal on each.
- Within each box, the slots should have symbols for harvesting, monitoring and sanctioning.
- Badges with animal drawings for each participant.
- The forest, with 100 trees.
- The ‘soil’, a brown mat on which the forest is placed.
- Harvesting forms. - 5 for the practice rounds, and 15 for the real rounds, plus a spare sheet.
- Debt cards. The cashier should have 100 cards.
- Cashier’s sheet 20 sheets (5 for practice, 15 for activity, +5 spares, dependent on country)
- Monitoring forms 3 for practice rounds, 15 for real rounds. (dependent on country)
- Sanctioning form 3 for practice rounds, 15 for real rounds (dependent on country)
- Observation forms - 40 (5 practice rounds, 15 real rounds, +8 spares, prepared for 2 observers)

S8 Fig. Decision box. Picture of animal with participant’s number

S9 fig. Inside decision box. participant’s number and pictures representing activities corresponding to given slot

S10 Fig. Income tokens

S11 Fig. Observation protocol

# Code Book for the Observation Protocol

***Instruction for the staff:*** Below, you will find examples of behaviors/quotes that correspond to each item of the observation protocol. This is NOT a complete list, just a guide, to give you the sense of what we are looking for. Participants’ behaviors are likely to be slightly different, so use your common sense to qualify them. For each item of the observation protocol we list several possible behaviors that we’ve see during our pilots (with examples of what participants might actually say in italics) – please code this behavior based on occurrence of any of these examples. For example, you code that participants share information about forest condition if they talk about anything related to the size of the change in forest cover.

**Participants share information about forest condition and/or its changes during the activity**

- Comments about the state of the forest
  - *the forest is looking good*
  - *the forest is small*
- Comments about changes in the forest cover
  - *when the forest is small, the regrowth is slower*
  - *we cut almost all of it*
  - *in this round the forest is smaller than before*

**Participants share information about their decisions (and/or their effects)**

- sharing information about individual harvesting decisions
  - *in the last round I harvested 4 trees*
  - *I didn’t really earn much in the last two rounds*
  - *Every round I cut 2 trees*
  - *This time I earned 8 tokens*
- sharing / combining information from group members about individual or group-level decisions
  - *together we harvested 10 trees*

*I know The Spider cut 3 trees and that’s more than I did***Participants (try to) coordinate tree harvesting**

- Proposing a common harvesting rule for two or more participants
  - *Next round let’s all harvest 2 trees per person*
  - *This time we can only harvest 23 trees in total as a group. How do we divide this allowance?*
- Persuading other participants to limit their harvesting
  - *I won’t cut any trees and you can harvest 3 trees this round but next round I will harvest 4 tress and you will harvest none.*
  - *If you harvest that many trees, the forest will die. You must limit your cutting.*
- Seeking for agreement on harvesting rules
  - *So do we have an agreement on the number of trees we can harvest?*

**Participants discuss ways to monitor whether arrangements / decisions are kept**

- Raising the issue of monitoring common arrangements
  - *So how do we know if all of us follow the harvesting rule?*
  - *I don’t trust some of you, I think we should check each other.*
- Proposing strategies to monitor other participants
  - *Everybody has to say aloud how many trees they harvest*
  - *Every round every participant monitors the person to their right*
  - *Every now and then I will monitor someone randomly.*
- Directly questioning other participants
  - *Tell me how many trees you harvested*

**Participants discuss ways to sanction others**

- Declaring sanctions individually
  - *Next round I will sanction the Spider.*
  - *Every round I will randomly sanction two of you until the harvesting falls below 10 trees per group per round.*
- Proposing common strategies to sanction other participants
  - *Next round everybody sanctions the Spider with two tokens.*
  - *Every round two Participants will be responsible for sanctioning offenders.*

**Participants declare adopting common strategies/ norms/ rules**

- Announcing common rules
  - *Next round everybody in the group can only harvest 1 tree (at least several participants nod or confirm verbally)*
- Announcing sanctions for disobedience
  - *If anyone harvests more than 3 trees, we will all sanction him/her*

**Participants praise other for obeying strategies/ norms/ rules**

- Praises to individual participants
  - *I’m so glad The Spider stopped harvesting like crazy and limited the cutting as suggested.*
  - *Spider, I thought you would be stubborn but you did well with the trees.*
- General positive comments
  - *I see we only harvested 12 trees as a group. That means we all obeyed the rule.*
  - *Look! We managed to stop forest degradation thanks to our limits.*

**Participants criticize or scold others for not obeying strategies/ norms/rules**

- Critique towards individual participants
  - *Spider, I know you are the one who harvested beyond the limit.*
  - *Stop cutting so much, you are greedy, Spider!*
  - *I thought we had an agreement but Mr Spider just had to do it his own way.*
- General negative comments
  - *We have a problem, somebody is cutting more than allowed. We can’t allow it.*
  - *Guys, stop cutting secretly, our forest is dying.*

**Do you think participants trust each other?**

- Direct statements suggesting lack of trust
  - *I don’t trust that bastard, he always cheats.*
  - *I want to see how much money you earned.*
  - *If you are so keen on forest conservation, why don’t you ever say aloud how many trees you harvested?*
- Nonverbal behaviors suggesting lack of trust (depending on the context)
  - Remaining silent during the general discussion or when asked questions
  - Trying to check the content of other peoples’ boxes

**How many of the Participants spoke?**

- How many participants decided to share their opinions?

**How much of the time (%) did each of the participants speak?**

- Try to estimate, how much time (% of the time in this round) each of the Participants spoke and write the result next to respective animal symbols. If someone kept silent, put 0% next to her/his animal symbol.

**Can you identify a leader among participants in this round? Please write who it is (the animal code).**

- Who do you think is the most influential participant?
- Who can affect other participants’ decisions/behaviors?
- Who is asked for advice or guidance?
- Who can stop fights and solve conflicts?

**In your opinion, how much did this group talk?**

For each round, try to estimate how much the group talked. One extreme would be that all participants remained silent for the whole round, the other extreme would be animated discussion with many (all) Participants talking, perhaps simultaneously.

**Other interesting behaviors** (please describe)

- Active resistance
  - *“I am not going to let you order me around.”*
  - *“I don’t have to tell you how much I earned”*
- Aggressive behaviors
  - *If you monitor me now, I will sanction you with all my tokens next round.*
- Confusion about rules of the activity
  - *I don’t understand how monitoring works.*
  - *What happens if I sanction with 3 tokens?*
  - *I didn’t know I would have to keep these tokens till the end of the activity.*
- And more…
