# Supplementary figures and images for: Conformity and tradition are more important than environmental values in constraining resource overharvest
Source: PLoS One. 2023 Feb 2;18(2):e0272366. doi: 10.1371/journal.pone.0272366 (PMC9894460; doi:10.1371/journal.pone.0272366)

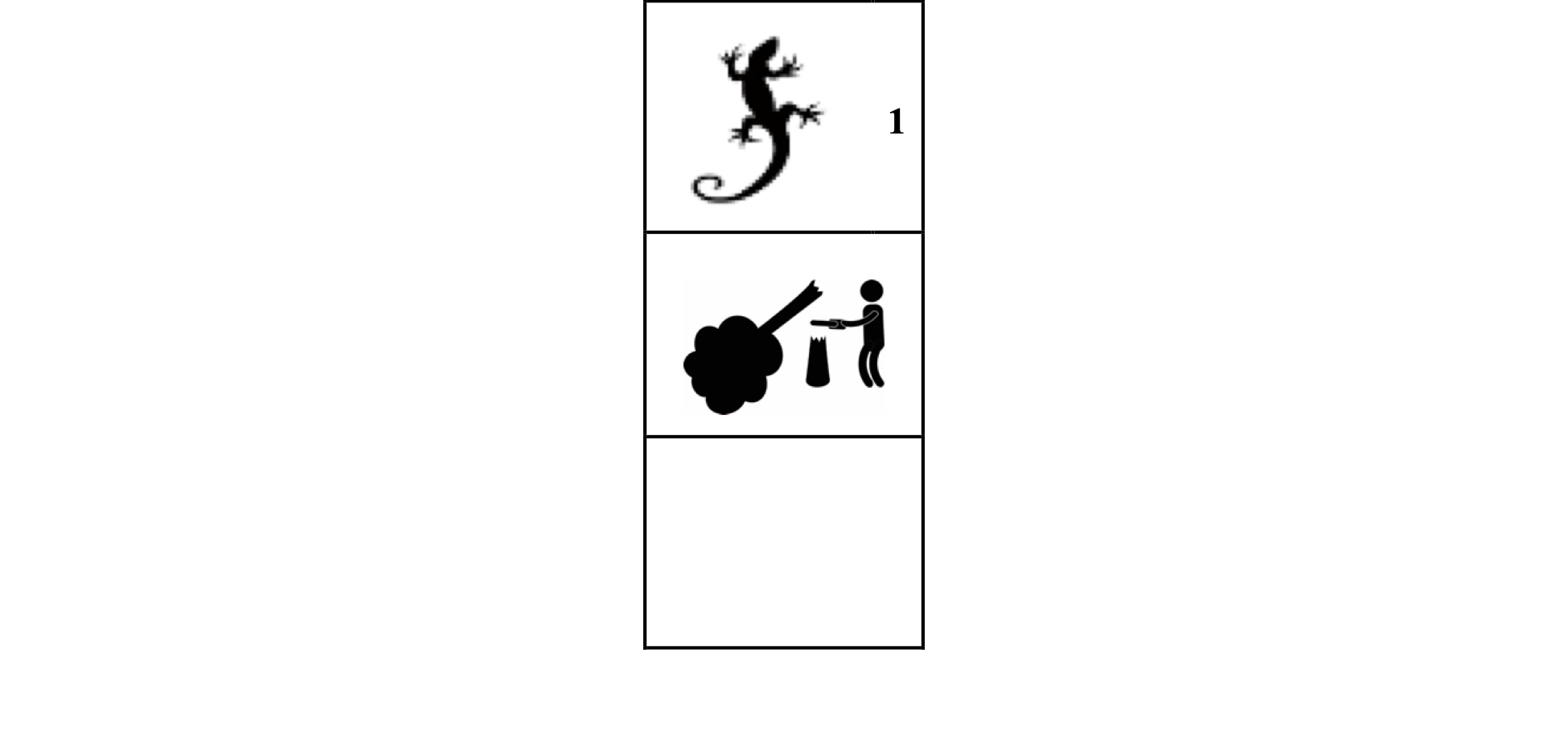

Supplement: S1 Fig — (TIF) [file pone.0272366.s002.tif]

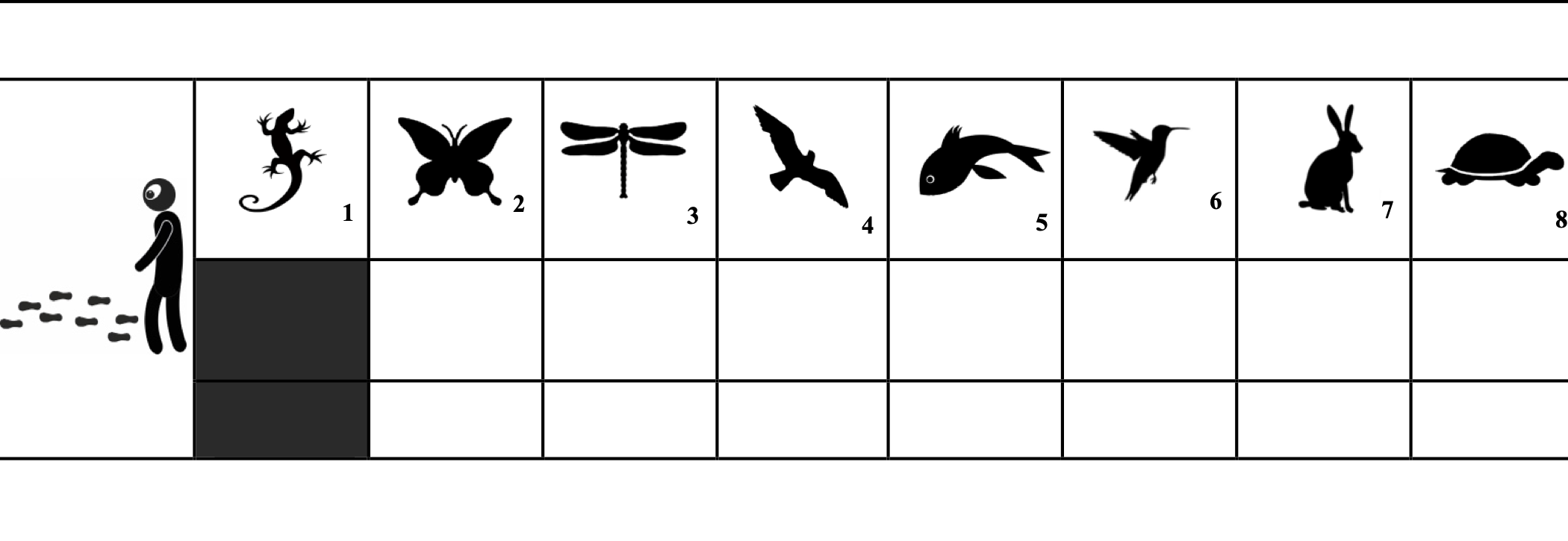

Supplement: S2 Fig — (TIF) [file pone.0272366.s003.tif]

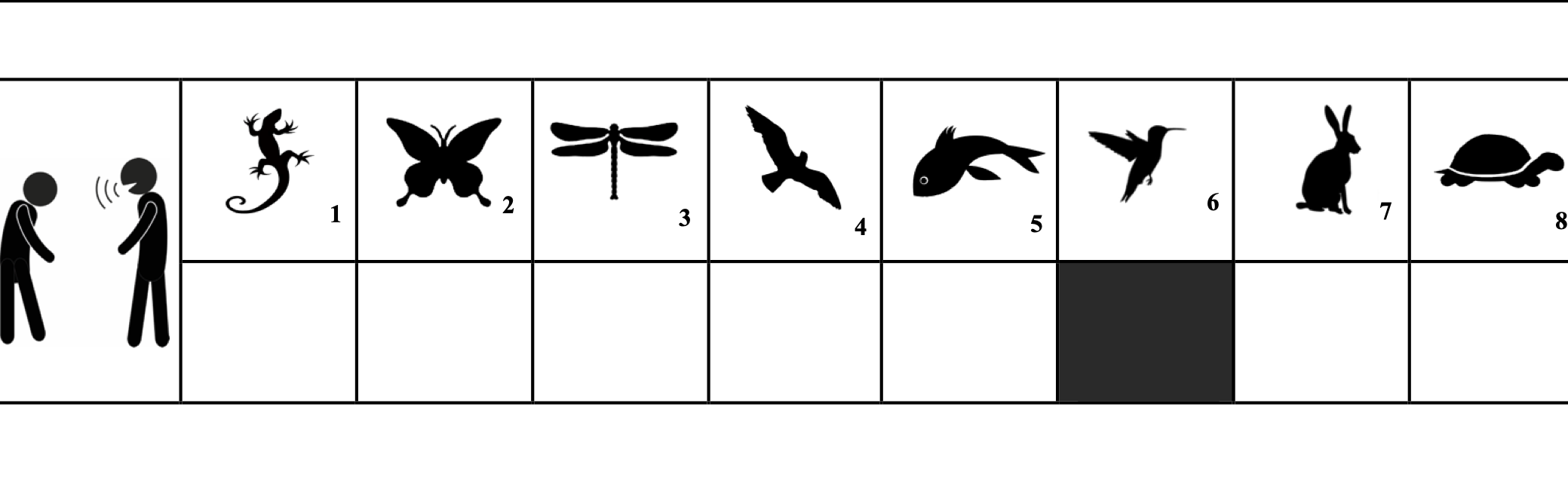

Supplement: S3 Fig — (TIF) [file pone.0272366.s004.tif]

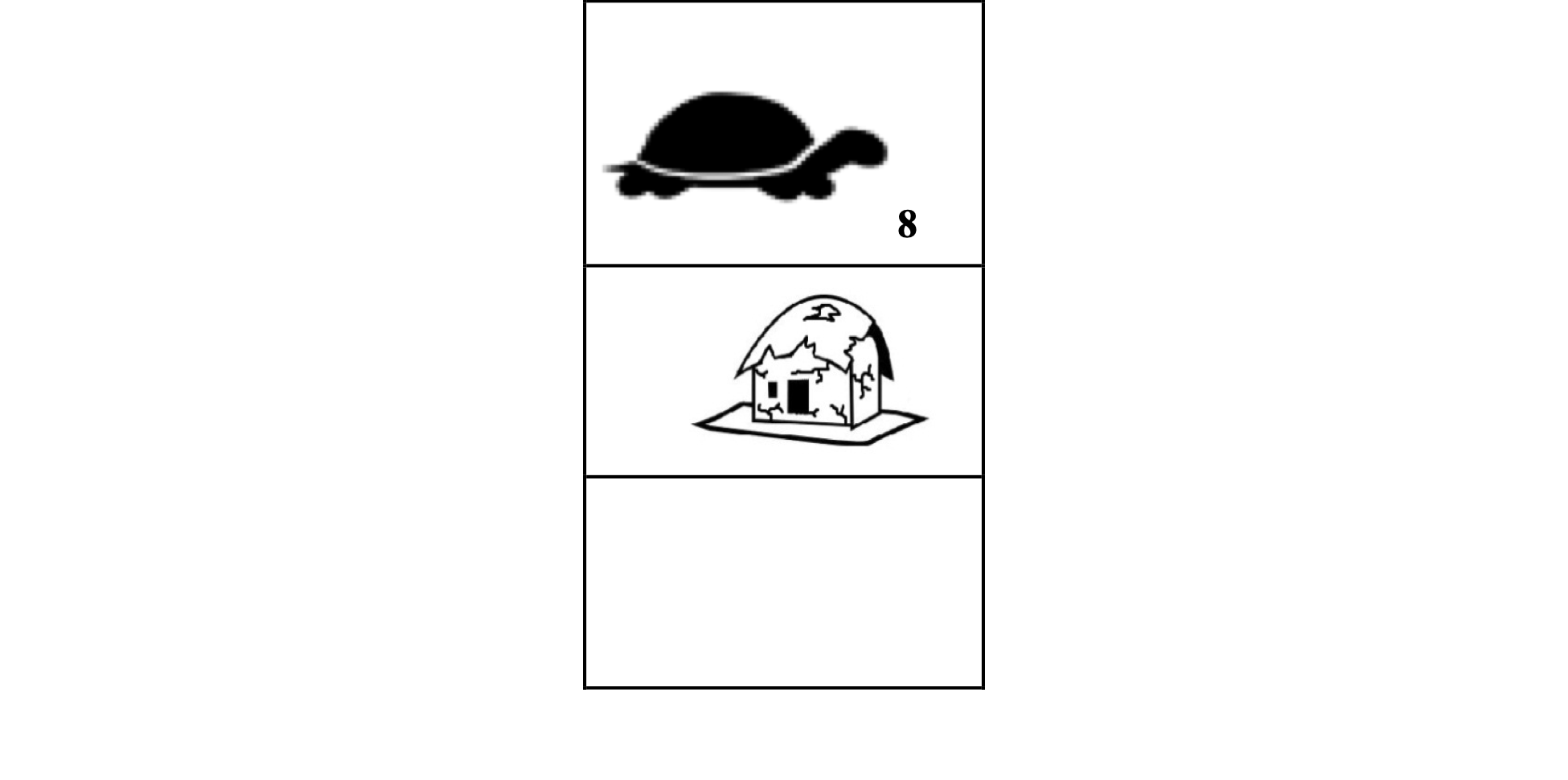

Supplement: S4 Fig — (TIF) [file pone.0272366.s005.tif]

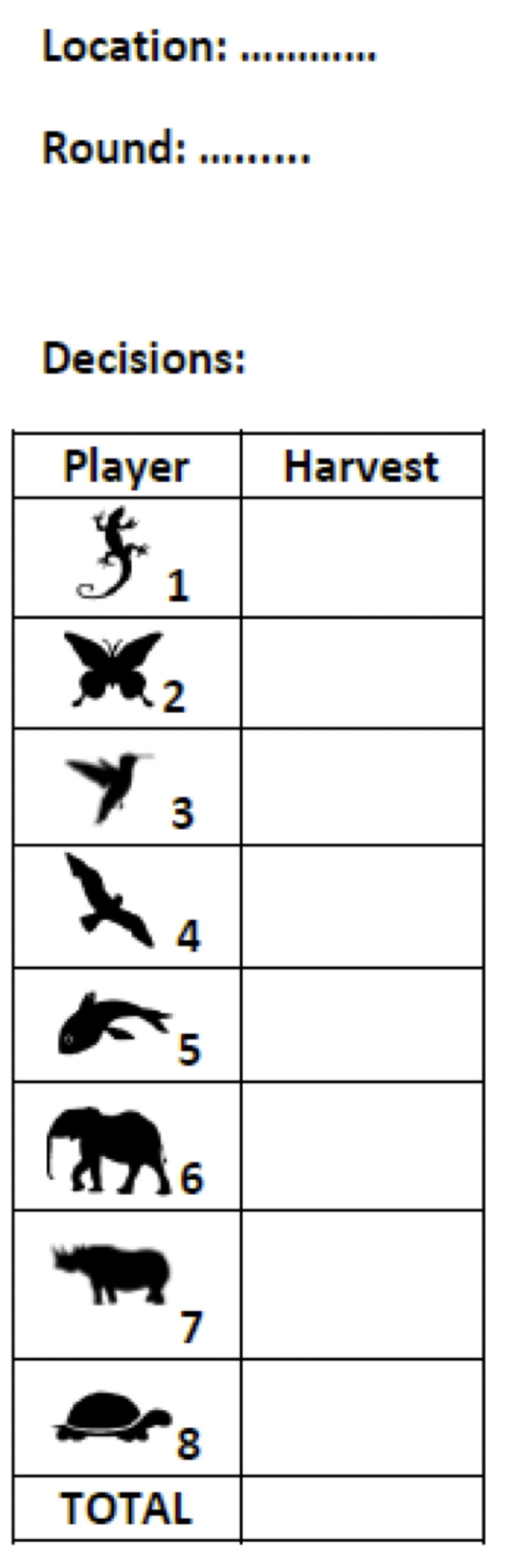

Supplement: S5 Fig — (TIF) [file pone.0272366.s006.tif]

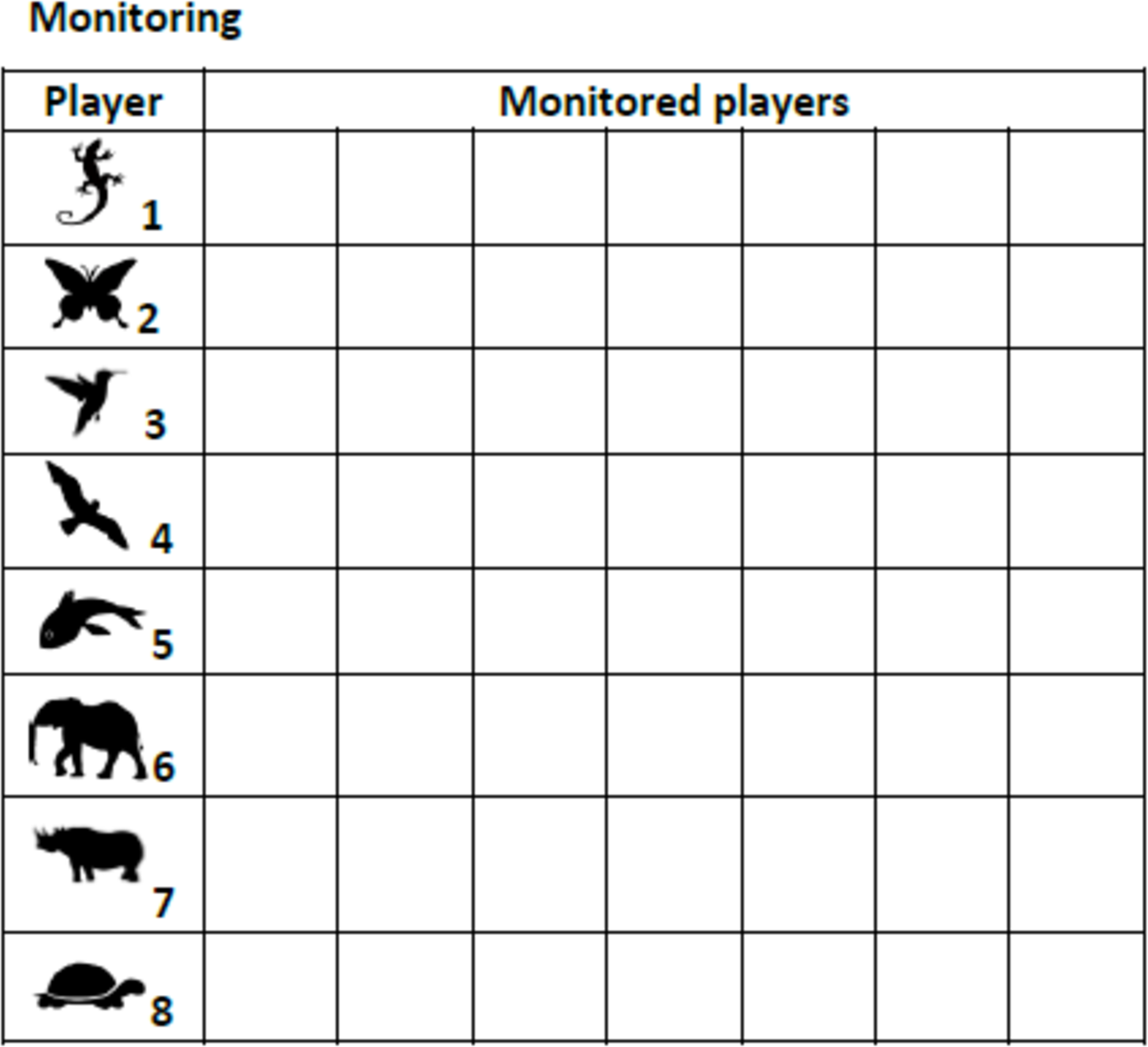

Supplement: S6 Fig — (TIF) [file pone.0272366.s007.tif]

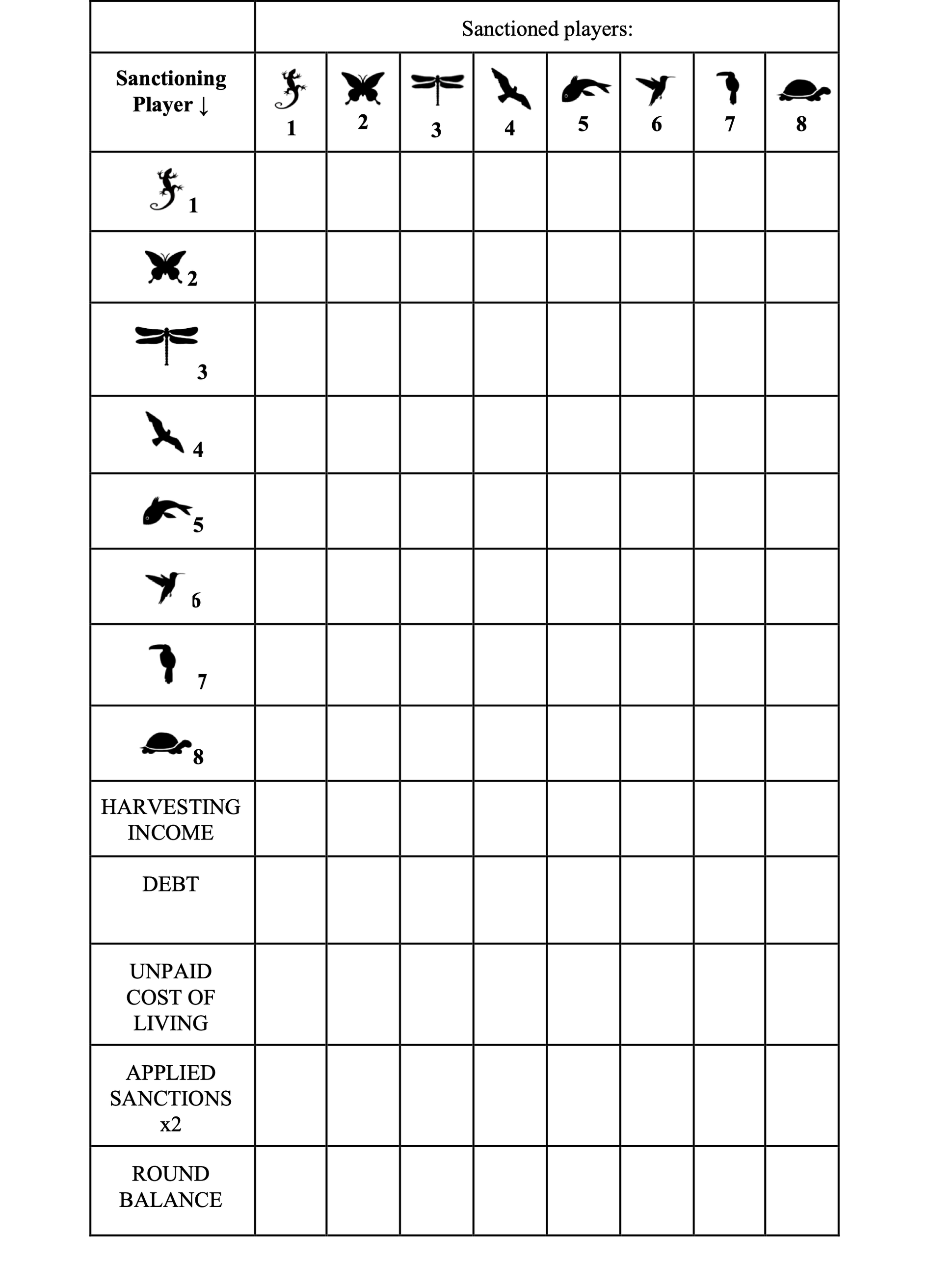

Supplement: S7 Fig — (TIF) [file pone.0272366.s008.tif]

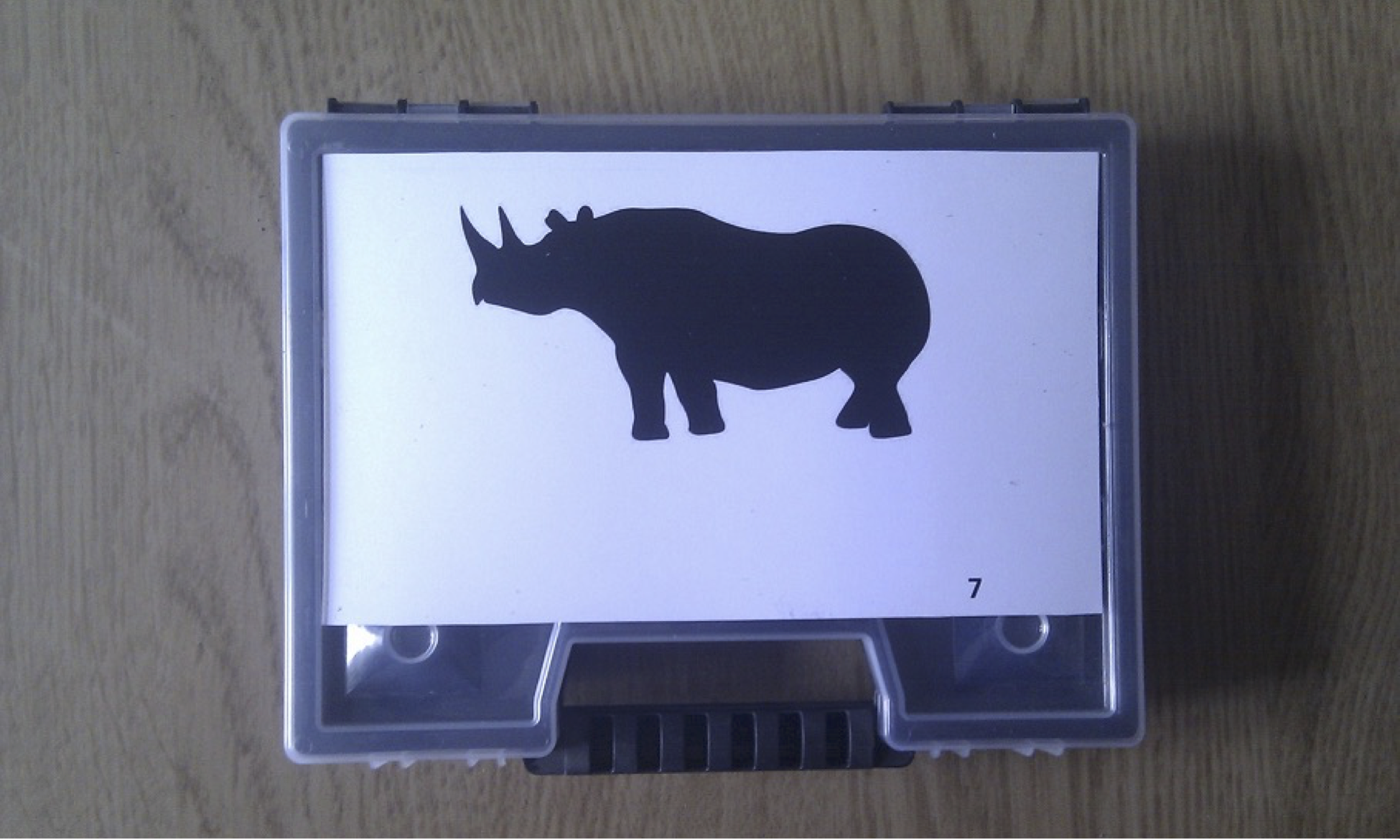

Supplement: S8 Fig — Picture of animal with participant’s number. (TIF) [file pone.0272366.s009.tif]

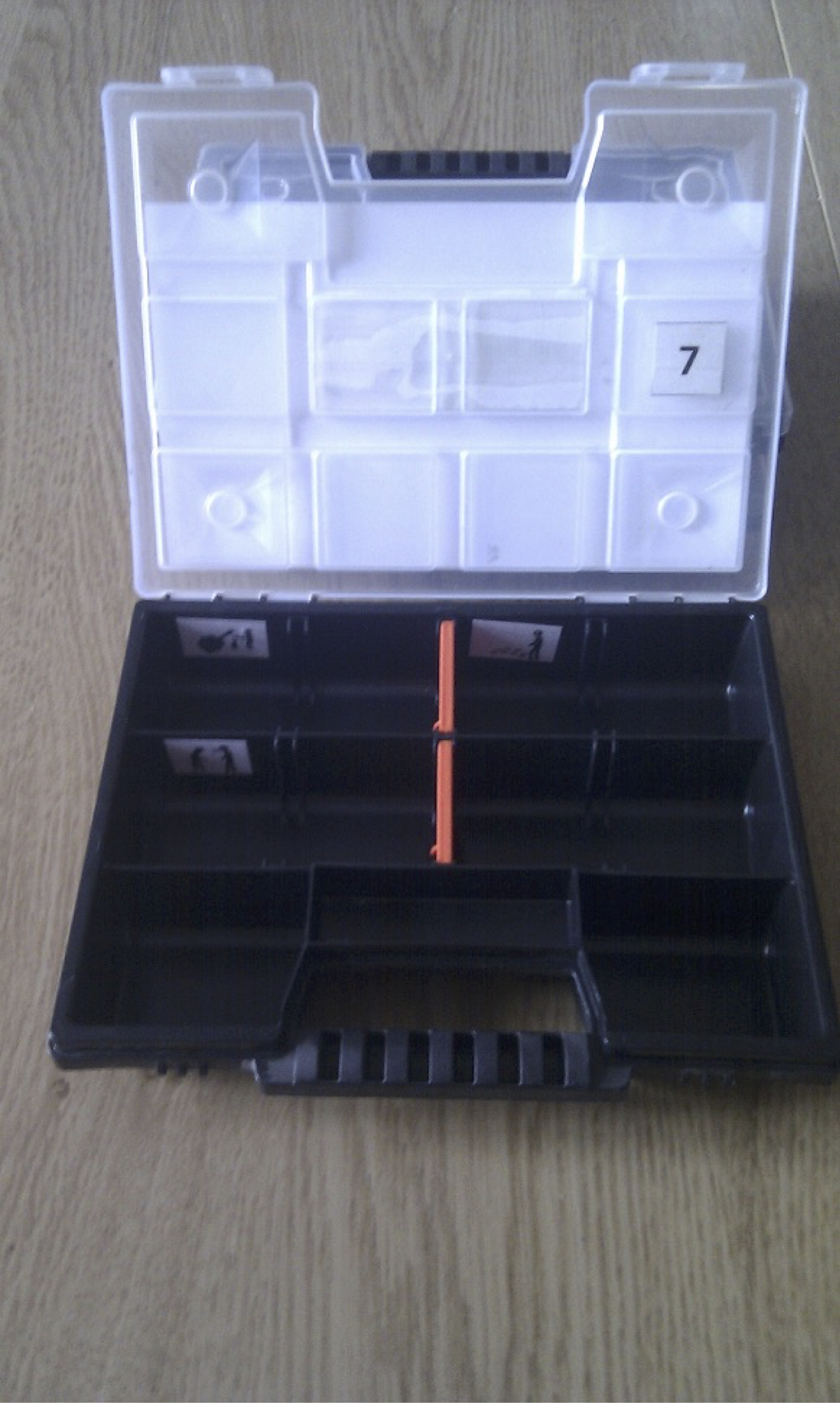

Supplement: S9 Fig — Participant’s number and pictures representing activities corresponding to given slot. (TIF) [file pone.0272366.s010.tif]

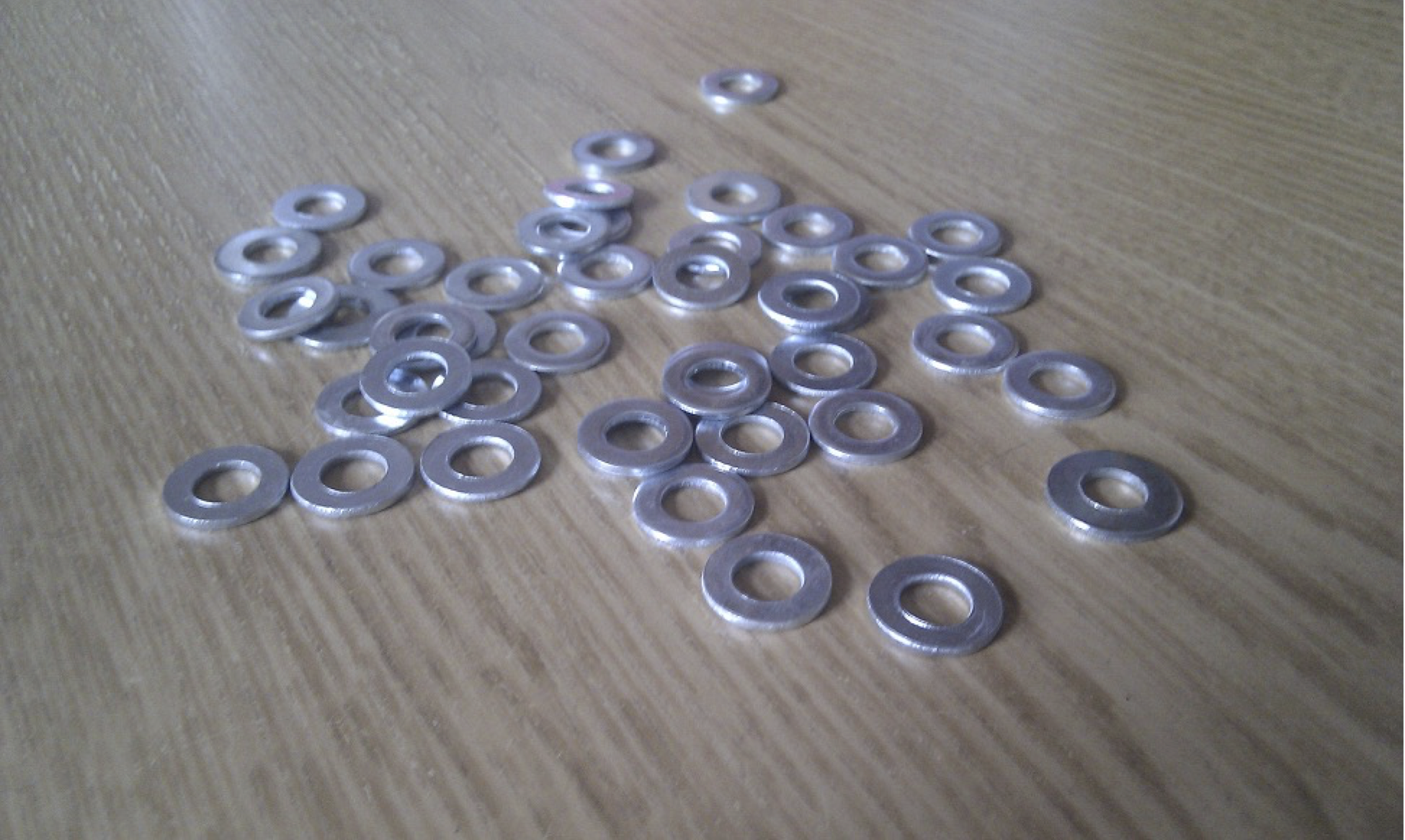

Supplement: S10 Fig — (TIF) [file pone.0272366.s011.tif]

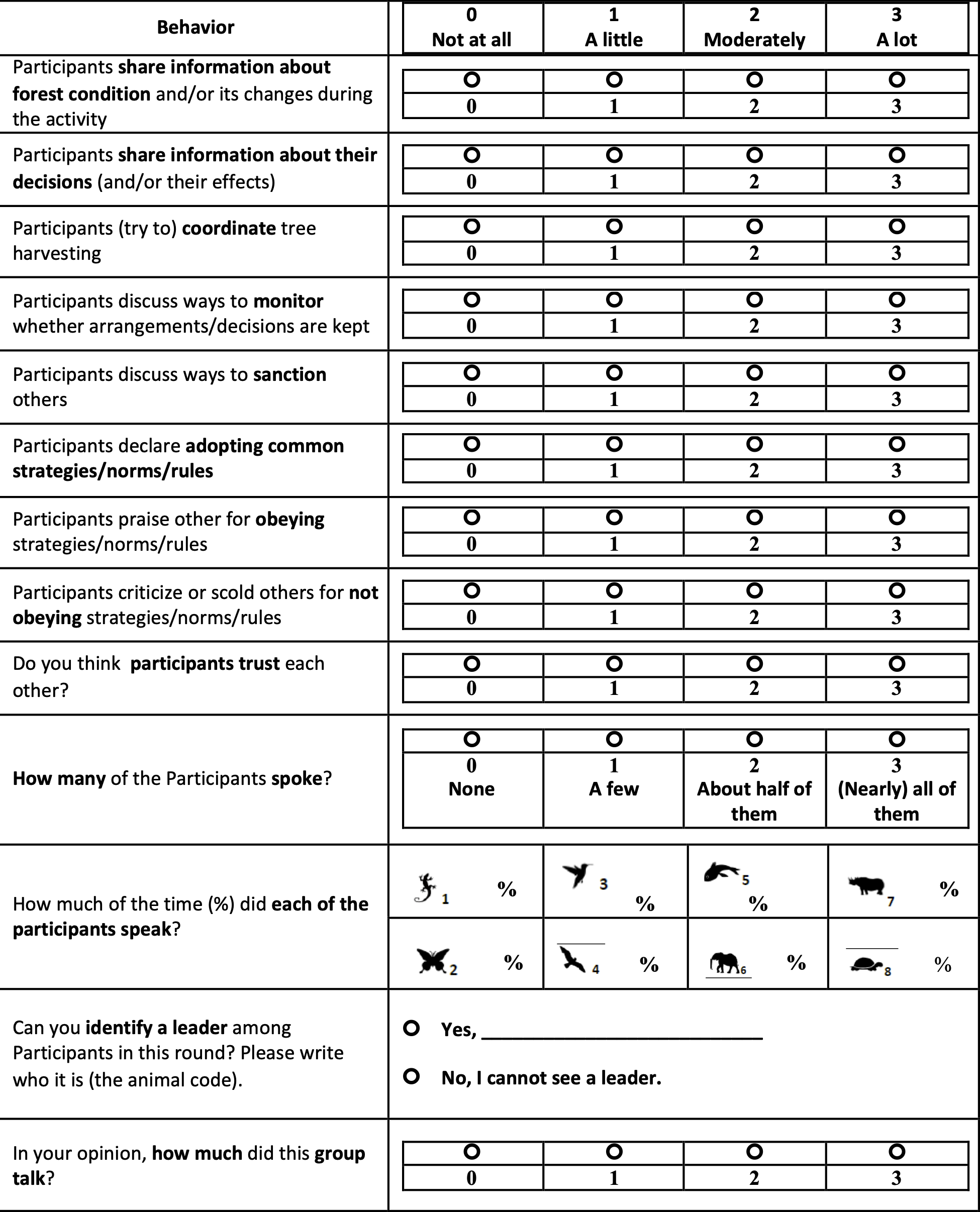

Supplement: S11 Fig — (TIF) [file pone.0272366.s012.tif]
