# Supplementary material for: Conformity and tradition are more important than environmental values in constraining resource overharvest
Source: PLoS One. 2023 Feb 2;18(2):e0272366. doi: 10.1371/journal.pone.0272366 (PMC9894460; doi:10.1371/journal.pone.0272366)
Supplement: Attachment — Submitted filename: Response to reviewers.docx [file pone.0272366.s013.docx]

Dr. Klain,

We are appreciative of your thoughtful comments on our paper, “Conformity and tradition are more important than environmental values in constraining resource overharvest.” We found your critiques thought-provoking and we have done our best to incorporate your suggestions into our set of revisions. We believe the result is a stronger paper. In order to clarify our changes, we have outlined your comments below and described the changes we’ve made in response to each. Reviewer comments are italicized and our responses are in regular text:

*I, however, see your research design as having a western, homo economicus approach to studying people outside of the “weird” cultures in which much of the theories underpinning political science and economics developed. I’m referencing Heinrich’s definition of “weird”: western, educated, industrialized, rich, democratic. The focus on individual values and differences at the individual level seem less relevant that a focus on identifying shared, collective values in your study sites. In the end, your data points to pro-social behavior driving decisions more than individually-oriented motivations driving decisions. In your set up and discussion of results, I think you ought to engage more with literatures that look beyond the individual, such as cultural ecosystem services, relational values and social valuation of the environment, e.g.:

Kenter, J. O., et al. (2016). Shared values and deliberative valuation: Future directions. Ecosystem Services, 21, 358–371.* [*https://doi.org/10.1016/j.ecoser.2016.10.006*](https://doi.org/10.1016/j.ecoser.2016.10.006) *Also, I encourage you to peruse:
Levine, J., Chan, K. M. A. & Satterfield, T. (2015). From rational actor to efficient complexity manager: Exorcising the ghost of Homo economicus with a unified synthesis of cognition research. Ecological Economics, 114, 22–32.* [*https://doi.org/10.1016/j.ecolecon.2015.03.010*](https://doi.org/10.1016/j.ecolecon.2015.03.010)

We appreciate these comments. It is quite true that we come from a political economy tradition based in rational choice theory (the “homo economicus” approach), though we hope that by expanding our inquiry to the role of values on resource governance we have stretched beyond those origins somewhat! And we have been careful to draw on a literature in social Psychology (the Schwartz theory of Values) which has been extensively tested outside of Western, Educated, Industrialized, Rich, and Democratic settings.

We have included new language in the paper, in the Introduction (first paragraph), and the literature review (“Values, Environment, and Resource Management,” first, fourth, and fifth paragraph that engage with these questions, outlining these thoughts in somewhat greater detail and connecting our research to the literature on shared values as you suggest.

The relationship between values held at the individual level (as measured by Schwartz) and the approach to shared value formation and elicitation (as outlined by Kenter and others) differ in some important (and perhaps paradoxical) ways. On the one hand, the Schwartz approach is methodologically individualistic in the sense that values are measured at the individual level, typically through survey instruments, and to the extent that values are measured at the group level by Schwartz and colleagues, those measured values are simply the aggregation of individual survey results. On the other hand, although the discursive approach to value elicitation and formation is definitively not based in the individual, much of the focus on the development and elicitation of values through discourse is related to decision-making process based in ecosystem services valuation. The notion of ecosystem services itself is controversial in some quarters because it too is perceived as too economistic, with scholars and practitioners essentially objecting to the idea that we can place a monetary value on ecosystems. We also have noted an additional criticism of this approach which is simply that ecosystem services valuation and similar processes are not widely used in many settings around the world, and even where discursive processes do not take place to elicit or develop shared values, policy-makers and resource users themselves hold values at the individual or group level; it seems important to us to examine whether these values have a measurable impact on resource management behaviors (hence our paper).

*Another interpretation of your results, which the Levine et al 2015 paper prompted me to recall, could be that people are efficiency maximizers. You evidence shows that identifying or not with pro-environmental values doesn’t explain behavior that well your study. Relying on tradition and conformity reduces cognitive burden and likely helps people maintain good relations in their small communities.*

This is a very interesting interpretation of our result, we would argue that this idea is complementary with our own interpretation, not necessarily a competing explanation; Collective action for conservation requires a shared understanding of the likely behaviors of other members of a particular community; if community members value tradition and conformity, they will likely have an easier time anticipating the actions of others because of a shared expectations around what is appropriate in a given situation. This might be both a way to efficiently manage complex situations (in a cognitive sense) and a way to enforce community norms (potentially by worsening interpersonal relationships in a predictable way when those norms are violated) in much the same way Ostrom and colleagues see institutions (rules, norms, and scripts) as ways to facilitate collective action. We have noted this helpful explanation in “Discussion” (paragraph 2).

*p. 3 last line: Scripts needs some introduction.*

Thank you, we have included a definition as a note.

*p. 6 Explain more in the intro about why a game microcosm sheds light on real world behavior. I now see that you reflect on this on p. 15 but I’d like to see some of this in the study’s set-up. I remain skeptical that how people behave in a game when they know they are being studied is a good proxy for real world behavior. A brief review of literature on how game behavior provides insight on real world behavior related to environmental management could convince me otherwise. It seems like quite a reductionist approach to understanding real-world forestry management and choices. It’s also not clear to me if deciding the amount of trees to harvest in your study sites is a collective or individual choice. My sense is that it’s likely more on the collective side, which doesn’t match your individual-centered research methods.*

These are important questions which touch on broad debates in Political Science and Economics, among other areas. We would never argue that simulations, games or experiments like the one we present here are a replacement for other empirical methods, but these types of simulations allow us to control for many types of endogeneity that threaten causal inference in studies using other empirical techniques (which often demonstrate correlation while attempting to investigate causation). We have included a longer description of the ways games, simulations, and experiments have been used to study resource governance and related questions in “Methods” (paragraph 2).

One interesting feature of our research design is that it allows us to examine both individual and collective choices and the link between the two; an important question we examine here is the extent to which values determine whether groups are able to make collective choices, i.e., whether individuals are more likely to stick to group agreements or to “cheat” and break agreements. In our simulation, harvesting decisions are made by individuals, but individuals within each group are free to communicate and therefore can (and often do) attempt to coordinate. However, like resource harvest in many remote settings, harvesting decisions are private and individual decisions are not immediately visible to other participants, so participants can “cheat” on those collective decisions (much like harvesting in real-world forest-dependent communities). Some groups are able to overcome these problems and act collectively while other are not—and of course our statistical analysis indicates that individuals with high levels of tradition and conformity are less likely to diverge from group agreements. We have inserted new language (paragraph 2 in “The forest management game” and paragraphs 9 and 10 in “Discussion”) to clarify these points.

*p.7. A map of your study site locations would be helpful.*

We have now added a map, currently Figure 1 on page 9. Thank you for this suggestion.

*p. 8 Please explain the Berns and Simpson 2009 reference a bit more. I would assume that people who work in extractive industries may spend lots of time in forests but not score high on conservation values.*

This is a fair point and we suspect is related to the reason we found no relationship between forest visitation and any of our outcomes of interest. We initially spent little time discussing this citation (or the forest exposure/ecological knowledge literature more broadly) because we found no relationship between forest visitation and environmental values or behavior but we have added some additional language to elaborate on the Berns and Simpson reference.

*p. 17 The last paragraph seems implicitly colonialist to me or at least vague about who is designing incentives and rules for resource governance. Also, “taking local values into account when designing incentives and rules for resource governance might lead to better outcomes” borders on banal. There’s plenty of justice-related arguments that already support this and decades of PES critiques that make this point repeatedly.*

We didn’t intend this paragraph to sound colonialist, and though we agree that the point is (or should be) banal, it perhaps bears repeating since much of the NGO and IGO community continues to pursue policies which seek to impose Western/Northern models on developing communities. That said, we recognize Dr. Klain’s point and have substantially restructured the last paragraph to remove these sentences.

We would again like to thank you for your helpful comments, and appreciate the opportunity to revise.

Sincerely

Glenn Wright
